# Functional relevance of nonsynonymous mutations in the HIV-1 *tat *gene within an epidemiologically-linked transmission cohort

**DOI:** 10.1186/1743-422X-4-107

**Published:** 2007-10-25

**Authors:** Haran Sivakumaran, Bin Wang, M John Gill, Brenda Beckholdt, Nitin K Saksena, David Harrich

**Affiliations:** 1 Division of Infectious Diseases and Immunology, Queensland Institute of Medical Research, Brisbane, Queensland, Australia; 2 School of Population Health, The University of Queensland, Brisbane, Queensland, Australia; 3 Retroviral Genetics Group, Centre for Virus Research, Westmead Millennium Institute, Westmead Hospital, The University of Sydney, Westmead, New South Wales, Australia; 4 Department of Medicine, University of Calgary, N.W. Calgary, Alberta, Canada

## Abstract

Here we investigated the nature and functional consequences of mutations in the HIV-1 *tat *gene within an epidemiologically-linked AIDS transmission cohort consisting of a non-progressing donor (A) and two normal progressing recipients (B and C). Multiple nonsynonymous mutations in the *tat *first exon were observed across time in all individuals. Some mutations demonstrated striking host specificity despite the cohort being infected with a common virus. Phylogenetic segregation of the *tat *clones at the time of progression to AIDS was also observed especially in recipient C. Tat clones supporting high levels of transactivation were present at all time points in all individuals, although a number of clones defective for transactivation were observed for recipient C in later time points. Here we show that the *tat *quasispecies in a linked transmission cohort diversify and evolve independently between hosts following transmission. It supports the belief that quasispecies variation in HIV-1 is a mechanism for selection towards defining a fitter gene variant that is capable of resisting the human immune system.

## Findings

HIV-1 transmission cohorts, where the donor, recipients and transmission histories are known, present an ideal opportunity to study the same virus in different immunological environments. Mutations in the *env *gene of HIV-1 have been the main focus in most epidemiologically-linked cohort studies of virus evolution [1,2], however relatively little in known about selection of mutations in the HIV-1 regulatory genes. One of the major regulatory genes of HIV-1 is *tat*, which encodes the viral transactivator of transcription known as Tat [3,4]. Originally discovered as an essential cofactor for efficient viral transcription, Tat is now ascribed to play diverse roles during AIDS pathogenesis [for reviews, see [5-7]]. Whilst there is no evidence to suggest that a specific Tat transactivation phenotype is selected during disease progression in a single host [8], little is known about the natural genetic and functional selection of diverse quasispecies of *tat *during transmission between hosts.

We attempted to determine if inter-host transmission of HIV-1 confers a selective pressure for Tat function in a unique epidemiologically-linked cohort of three individuals [1,9]. The cohort consisted of a long-term non-progressor (donor A) who transmitted HIV-1 to two recipients (B and C) via blood transfusion. The recipients subsequently developed AIDS and progressed normally, with recipient C recently dying from an AIDS-related illness following rapid progression around the time of death. Infected peripheral blood mononuclear cells (PBMCs) were collected from the individuals at various time points and the integrated first-exon *tat *sequences were amplified from these cells.

**Figure 1:**
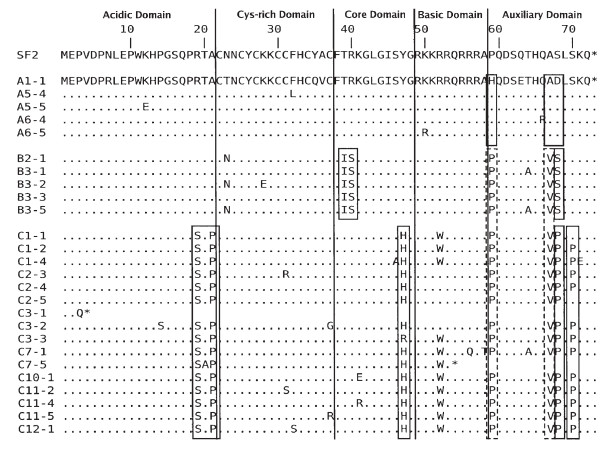
**Amino acid alignment of cohort Tat clones**. The sequenced cohort Tat clones are aligned against clone A1-1. A dot represents amino acid identity at that position; an asterisk represents a stop codon. Tat domains as described by [19] are separated by vertical lines, individual-specific substitutions are indicated by solid boxes and substitutions common to recipients B and C by dashed boxes. The amino acid sequence of one-exon Tat from HIV-1 clone SF2 is shown for comparison. The nucleotide sequences of these clones are available from GenBank ([GenBank:EU184659] – [GenBank:EU184684]).

**Figure 2:**
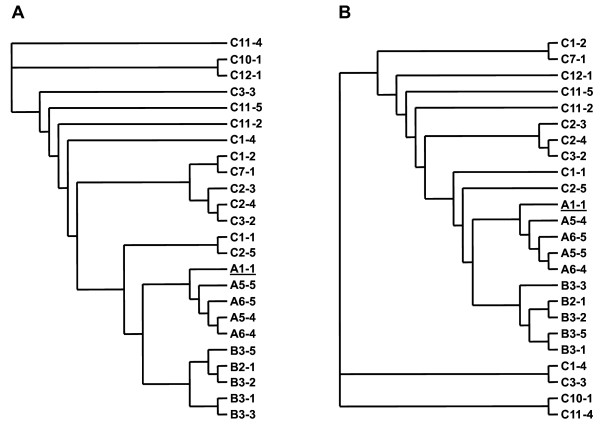
**Phylogenetic analysis**. Neighbour-joining phylogenetic reconstruction of *tat *clones based on nucleotide (A) and peptide (B) sequences. The differences in the tree topologies suggest nonsynonymous evolution of *tat *in each host. Donor A's clone A1-1 is underlined in both cladograms.

Multiple first-exon *tat *sequences were amplified by nested PCR from the PBMCs of the cohort members at 5 time points from donor A, 4 time points from recipient B and 12 time points from recipient C. (Refer to additional file 1: detailed methods.) These amplicons were subsequently cloned into expression vectors and a total of 89 *tat *clones were generated. Twenty-six unique clones were identified after comparison of amino acid sequences ([GenBank:EU184659] – [GenBank:EU184684]). These unique clones were aligned against the most prevalent clone from donor A (clone A1-1), which revealed the presence of multiple amino acid substitutions in all individuals (Figure1). Host-specific mutations are highlighted by solid boxes in Figure 1 whereas mutations common between hosts are marked with dashed boxes. Attestation of these changes was also visualised using phylogenetic reconstruction of the *tat *clones using both nucleotide and peptide sequences. The nucleotide (Figure 2A) and peptide (Figure 2B) topologies were distinct suggesting that the nonsynonymous changes in the *tat *genes may have some bearing on the genetic relationship between *tat *clones from a single individual and may carry functional relevance, as confirmed herein.

Tat proteins from the donor A clones were generally comprised of previously observed amino acid residues as described in the Los Alamos HIV Sequence Database [10,11]. Residues in the donor A clones considered infrequent or rare were E12, L32, and R66, as well as residues H59 and D68, which were both common to all donor A clones. The D68 residue has not been previously described and was not observed in Tat clones of recipients B or C, which possessed the commonly found S68 or P68 residues. Recipient B's host-specific mutations (compared to clone A1-1) were T39I, R40S and D68S. Recipient C's host-specific mutations, in contrast, were R19S, A21P, Y47H (except clone C3-3), D68P and S70P (except clones C1-1 and C2-5). The substitutions H59P and A67V were seen in all clones from recipients B and C (dashed boxes in Figure 1) but not in any of the clones from donor A. Thus distinct nonsynonymous mutations were observed in the Tat clones from all cohort members that segregated in a host-specific manner as well as two mutations that showed common specificity to the transmission recipients. The specificity of these mutations are consistent with host-driven evolution of the *tat *quasispecies in each cohort member.

There were considerable differences in sequence diversity between Tat clones from the donor and the two recipients. Donor A clones showed less diversity in amino acid sequences compared to the recipients, whereas recipient B clones were less diverse than clones from recipient C. Interestingly, none of the amino acid mutations identified in the donor were observed in either of the recipients, who share more nonsynonymous mutations between them compared to their common donor.

**Figure 3:**
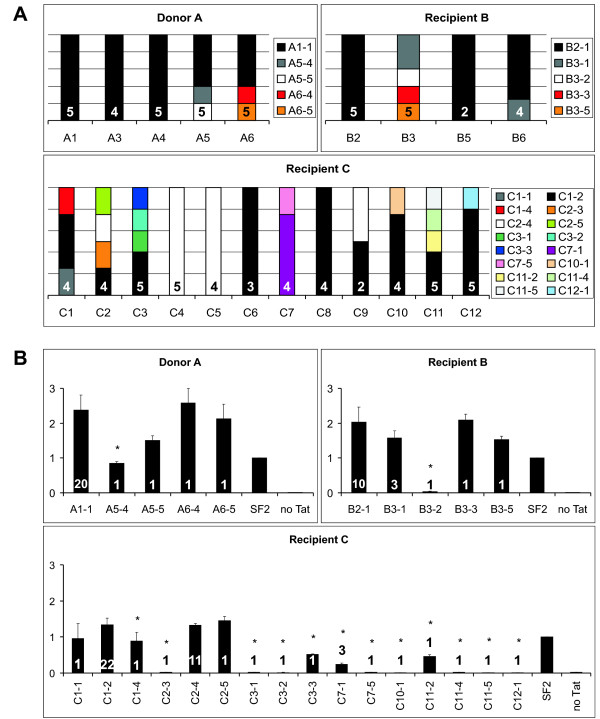
**Composition, variation, and activity of the cohort's *tat *quasispecies over time**. (A) Multiple one-exon *tat *clones from donor A, recipient B and recipient C were sequenced and their amino acid sequences were compared at each time point (represented as columns). Identical amino acid sequences were classed together as clones and are represented above as boxes within the columns. The numbers within the columns indicate the total number of *tat *clones successfully sequenced for each time point. See Figure 1 for the clones' amino acid sequences. (B) Relative transactivation abilities of the cohort *tat *clones. Columns are transactivated luciferase output normalised against constitutive β-galactosidase output and expressed relative to a positive control for transactivation (the SF2 clone of one-exon *tat*). The values at the bases of the columns indicate the number of times that particular Tat amino acid sequence was scored in the entire sample set. An asterisk indicates *p *< 0.01 for the null hypothesis compared to clone A1-1. Results are means and standard deviations of three independent experiments.

Further, demonstration of these host-specific differences in viral quasispecies is depicted in Figure 3A, which shows the scoring of the different Tat amino acid sequences in the *tat *quasispecies over time. Figure 3A depicts each time point as stacked columns representing the composition of the *tat *quasispecies based on amino acid sequence. For example, time point A5 shows that three of five sequenced Tat clones were identical to clone A1-1 with the remaining two clones identified as clones A5-4 and A5-5. The data identify dominant *tat *clones present in all three individuals: clone A1-1 for donor A, clone B2-1 for recipient B and clones C1-2 and C2-4 for recipient C. These clones were present in most of the time points (or all of the time points for donor A) within the respective individual but were not seen in any other individual. Overall, despite differences in HIV-1 genetic variability in each member of the cohort, there was considerable stability in the quality of mutations over time in each individual.

The transactivation abilities of each individual's unique Tat clones were assessed using a luciferase reporter assay. The luciferase reporter contains the HIV-1 LTR upstream of the *luc *gene meaning that specific binding of Tat to an RNA structure (the transactivation response element, or TAR) in the LTR drives powerful expression of luciferase. Only the protein expressed from the first exon of *tat *is required to fully transactivate the LTR [12], thus we tested the first exons of the *tat *clones in the assay. The transactivated luciferase output of each one-exon *tat *clone are represented in Figure 3B as fold activation over a control one-exon *tat *gene from the SF2 isolate of HIV-1. Transfection efficiencies were normalised with a β-galactosidase expression plasmid. This accounts for variations in plasmid amounts but not, however, for variations in Tat clone expression levels or protein stability. Clones from donor A demonstrated two- to three-fold transactivation over SF2 Tat with all but clone A5-4 showing no significant difference (*p *> 0.01) compared to clone A1-1. Similarly for recipient B, all but clone B3-2 showed no difference in transactivation compared to A1-1. The low values for A5-4 and B3-2 are attributable to substitutions in the cysteine-rich domain of Tat (F32L and K28E, respectively), a critical region for transactivation and intramolecular bonding [13,14].

The Tat clones from recipient C possessed the widest diversity of transactivation function. Twelve of the sixteen unique clones showed significantly less (*p *< 0.01) transactivation abilities compared to A1-1 (denoted by asterisks in Figure 3B). The general attenuation seen in all of recipient C's Tat clones is most likely due to two mutations, Y47H and R52W, located in the highly conserved core and basic domains (respectively) of Tat. The core domain mutation has been reported to suppress but not eliminate transactivation ability [15-17], and R52 participates in the binding of Tat to TAR and is involved in the nuclear localisation of Tat [18,19]. The strong or total suppression of transactivation abilities observed in many of the recipient C clones is due to various mutations in the cysteine-rich and core domains or, in the case of clones C3-1 and C7-5, due to premature stop codons (Figure 1).

It is interesting, and apparently paradoxical, to note that many of the defective Tat clones in recipient C appeared at later time points around the time of rapid progression. It is possible that loss of viral transactivation ability may be required for rapid disease progression in this particular individual. Alternatively, the detection of inactive *tat *mutants could have been enhanced through the sampling of *tat *genes from lower amounts of PBMCs at these later time points, especially CD4^+ ^T cells and other HIV-1 reservoirs (see additional file 2: cohort data). However it should be stressed that fully active Tat could consistently be detected in recipient C at nearly all time points and that these defective Tat mutants were not dominant in the quasispecies population (Figure 3A). In general our results suggest that the majority of Tat clones from donor A and recipients B and C activated the HIV-LTR similarly to donor A's clone A1-1, whilst most of the latter time-point clones from recipient C were attenuated.

The evidence presented here demonstrate the selection of multiple nonsynonymous mutations in *tat *in a unique epidemiologically-linked cohort following transmission of HIV-1. Comparisons of the relative transactivation abilities of the Tat clones indicated that the donor and recipients had signature *tat *genes that conferred strong transactivation potential. While these experiments do not link a *tat *transactivation mutation to disease progression, it remains possible that alternative Tat functions may contribute to disease progression and that these may be subject to selective pressures during transmission independent of transactivation function. Quasispecies modulation *in vivo *is vital to the survival of HIV-1 as well as the functional selection of a dominant variant that is capable of counteracting neutralisation by the host immune system.

## Competing interests

The author(s) declare that they have no competing interests.

## Authors' contributions

HS and BW performed the experiments. BB and MJG provided the samples from the cohort. NKS and DH supervised the project, and all authors contributed to the text.

## Supplementary Material

Additional file 1 Detailed methods. Detailed description of the study methodologies. Click here for file

Additional file 2 Cohort data. Viral loads, CD4^+ ^and CD8^+ ^cell counts of the cohort at each time point. Click here for file

## References

[B1] Mikhail M, Wang B, Lemey B, Beckthold B, Vandamme A, Gill JM, Saksena NK (2005). Role of viral evolutionary rate in HIV-1 disease progression in a linked cohort. Retrovirology.

[B2] Song JZ, Wang B, Ge YC, Dwyer DE, Cunningham AL, Saksena NK (1999). Significance of plasma and peripheral blood mononuclear cell derived HIV-1 sequences in establishing epidemiologic linkage between two individuals multiply exposed to HIV-1. Microb Pathog.

[B3] Arya SK, Guo C, Josephs SF, Wong-Staal F (1985). Trans-activator gene of human T-lymphotropic virus type III (HTLV-III). Science.

[B4] Sodroski J, Patarca R, Rosen C, Wong-Staal F, Haseltine W (1985). Location of the trans-activating region on the genome of human T-cell lymphotropic virus type III. Science.

[B5] Huigen MC, Kamp W, Nottet HS (2004). Multiple effects of HIV-1 trans-activator protein on the pathogenesis of HIV-1 infection. Eur J Clin Invest.

[B6] Harrich D, Hooker B (2002). Mechanistic aspects of HIV-1 reverse transcription initiation. Rev Med Virol.

[B7] Pugliese A, Vidotto V, Beltramo T, Petrini S, Torre D (2005). A review of HIV-1 Tat protein biological effects. Cell Biochem Funct.

[B8] Delassus S, Meyerhans A, Cheynier R, Wain-Hobson S (1992). Absence of selection of HIV-1 variants in vivo based on transcription/transactivation during progression to AIDS. Virology.

[B9] Mikhail M, Wang B, Lemey P, Beckholdt B, Vandamme AM, Gill MJ, Saksena NK (2005). Full-length HIV type 1 genome analysis showing evidence for HIV type 1 transmission from a nonprogressor to two recipients who progressed to AIDS. AIDS Res Hum Retroviruses.

[B10] Leitner T, Foley B, Hahn B, Marx P, McCutchan F, Mellors J, Wolinsky S, Korber B (2005). HIV Sequence Compendium 2005.

[B11] HIV Sequence Database. http://www.hiv.lanl.gov/content/hiv-db/mainpage.html.

[B12] Seigel LJ, Ratner L, Josephs SF, Derse D, Feinberg MB, Reyes GR, O'Brien SJ, Wong-Staal F (1986). Transactivation induced by human T-lymphotropic virus type III (HTLV III) maps to a viral sequence encoding 58 amino acids and lacks tissue specificity. Virology.

[B13] Rice AP, Carlotti F (1990). Structural analysis of wild-type and mutant human immunodeficiency virus type 1 Tat proteins. J Virol.

[B14] Koken SE, Greijer AE, Verhoef K, van Wamel J, Bukrinskaya AG, Berkhout B (1994). Intracellular analysis of in vitro modified HIV Tat protein. J Biol Chem.

[B15] Verhoef K, Berkhout B (1999). A second-site mutation that restores replication of a Tat-defective human immunodeficiency virus. J Virol.

[B16] Verhoef K, Koper M, Berkhout B (1997). Determination of the minimal amount of Tat activity required for human immunodeficiency virus type 1 replication. Virology.

[B17] Hooker CW, Scott J, Apolloni A, Parry E, Harrich D (2002). Human immunodeficiency virus type 1 reverse transcription is stimulated by tat from other lentiviruses. Virology.

[B18] Hauber J, Malim MH, Cullen BR (1989). Mutational analysis of the conserved basic domain of human immunodeficiency virus tat protein. J Virol.

[B19] Kuppuswamy M, Subramanian T, Srinivasan A, Chinnadurai G (1989). Multiple functional domains of Tat, the trans-activator of HIV-1, defined by mutational analysis. Nucleic Acids Res.

