# Supplementary material for: Functional relevance of nonsynonymous mutations in the HIV-1 tat gene within an epidemiologically-linked transmission cohort
Source: Virol J. 2007 Oct 25;4:107. doi: 10.1186/1743-422X-4-107 (PMC2174444; doi:10.1186/1743-422X-4-107)
Supplement: Additional file 1 — Detailed methods. Detailed description of the study methodologies. [file 1743-422X-4-107-S1.pdf]

Detailed methods

Cell culture and transfections

HeLa cells were cultured in RPMI 1640 medium supplemented with 100 U/ml penicillin, 100 μl/ml streptomycin and 10% (v/v) newborn calf serum (Invitrogen Corporation, Carlsbad, California, U.S.A.) and incubated at 37°C under a humidified atmosphere of 5% CO_2. Transfections were performed using Lipofectamine 2000 transfection reagent (Invitrogen Corporation) according to the manufacturer's instructions.

Plasmid constructs

Near full-length proviral cDNA were PCR-amplified from the individuals' peripheral blood mononuclear cells (PBMCs) at each time point as previously described [1]. First-exon *tat* sequences were amplified from the cDNA via a nested PCR, firstly using the external primers 5'-AGGAGTGGAAGCCATAATAA-3' and 5'-ATTGCCACTGTCTTCTGCTGT-3', and then the internal primers 5'-<u>ACAAGTTTGTACAAAAAAGCAGGCT</u>ATGGAGCCAGTAGAT-3' and 5'-<u>ACCACTTTGTACAAGAAAGCTGGGT</u>ATGCTACTACTAATG-3'. Amplicons were cloned into vector pDONR201 (via *att*B sites, underlined in the internal primer sequences) and then into the expression vector pcDNA-DEST40 using Gateway cloning technology (Invitrogen Corporation) in a manner that places a stop codon immediately after the first-exon *tat* sequence. Up to five clones per time point were isolated, totalling 89 clones. Clones were sequenced using dye terminators version 3.1, translated and aligned against HIV-1 clone SF2 [2] Tat using the European Molecular Biology Open Source Software Suite programs TRANSEQ and SHOWALIGN [3]. Unique clones from each individual were then chosen for further study. The sequences of these unique clones are available from the GenBank database with accession numbers EU184659 – EU184684.

The one-exon *tat* sequence from SF2 was similarly cloned into pcDNA-DEST40 and used as a control for the transactivation study. The study was performed using a luciferase assay system,

which included a luciferase reporter plasmid constructed by cloning the *Hind*III to *Bam*HI fragment of the SF2 long terminal repeat (LTR) into vector pGL3-basic (Promega Corporation, Madison, Wisconsin, U.S.A.) to form pGL3-LTR. A CMV immediate-early promoter-driven β-galactosidase expression plasmid (pCMV-*lac*Z) was also included as a control for transfection efficiencies.

Phylogeny analysis

The alignment of *tat* gene sequences of HIV-1 strains with reference subtypes was done using CLUSTAL-W from the GCG package, followed by minor manual adjustments considering protein sequences. Gaps (insertions/deletions) were stripped prior to phylogenetic and bootscan analyses, using the BioEdit Sequence Alignment Editor (version 5.9.0, T. Hall, North Carolina State University, Raleigh, NC). Phylogenetic analysis was done using the PHYLIP package (version 3.5c; J. Felsenstein, University of Washington, Seattle, WA) based on default parameters. Pairwise nucleotide evolutionary distances were calculated using the Kimura two-parameter model with a transition/transversion ratio of 2.0. Phylogenetic trees were then constructed using the neighbour-joining method. The reliability of the nodes was confirmed by bootstrap analysis with 100 replicates using Seqboot and Consense. Bootstrap values of ≥ 70% were considered to be significant for assignment of parenthood [4].

Transactivation assay

HeLa cells were co-transfected in 24-well plates with Lipofectamine 2000 and 300 ng each of individual's *tat* expression plasmids, pGL3-LTR and pCMV-*lac*Z. The SF2 *tat* expression plasmid was similarly transfected as a control. Cells were harvested and lysed 24 h post-transfection by incubating in Glo Lysis Buffer (Promega Corporation) and manually aspirating with a pipette. Lysates were assayed for luciferase activity using the Steady-Glo luciferase assay kit (Promega Corporation) as per the manufacturer's instructions. Light output was quantitated on a Wallac TriLux MicroBeta counter (PerkinElmer, Inc., Wellesley, Massachusetts, USA). Lysates were

assayed for β-galactosidase activity using the chlorophenol red-β-D-galactopyranoside (CPRG)-based assay [5].

Statistical analyses

The null hypothesis for the transactivation study was that the relative luciferase output of cells transfected with each individual's *tat* clone was equal to the relative luciferase output of clone A1-1. The hypothesis was tested using Student's *t*-test against a two-tailed distribution assuming homoscedastic variances.

References

1. Gill MJ, Towns D, Allaire S, Meyers G: **Transmission of human immunodeficiency virus through blood transfusion: the use of lookback and traceback approaches to optimize recipient identification in a regional population.** *Transfusion* 1997, **37:**513-516.

2. Sanchez-Pescador R, Power MD, Barr PJ, Steimer KS, Stempien MM, Brown-Shimer SL, Gee WW, Renard A, Randolph A, Levy JA, et al.: **Nucleotide sequence and expression of an AIDS-associated retrovirus (ARV-2).** *Science* 1985, **227:**484-492.

3. Rice P, Longden I, Bleasby A: **EMBOSS: the European Molecular Biology Open Software Suite.** In *Trends Genet*, vol. 16. pp. 276-277; 2000:276-277.

4. Mikhail M, Wang B, Lemey B, Beckthold B, Vandamme A, Gill JM, Saksena NK: **Role of viral evolutionary rate in HIV-1 disease progression in a linked cohort.** *Retrovirology* 2005, **2:**41.

5. Eustice DC, Feldman PA, Colberg-Poley AM, Buckery RM, Neubauer RH: **A sensitive method for the detection of beta-galactosidase in transfected mammalian cells.** *Biotechniques* 1991, **11:**739-740, 742-733.
