# Supplementary material for: Functional relevance of nonsynonymous mutations in the HIV-1 tat gene within an epidemiologically-linked transmission cohort
Source: Virol J. 2007 Oct 25;4:107. doi: 10.1186/1743-422X-4-107 (PMC2174444; doi:10.1186/1743-422X-4-107)
Supplement: Additional file 2 — Cohort data. Viral loads, CD4+ and CD8+ cell counts of the cohort at each time point. [file 1743-422X-4-107-S2.pdf]

Cohort data

Viral loads, $CD4^+$ and $CD8^+$ cell counts of the cohort at each time point.

Time Point	Year	Viral Load (copies/ml)	$CD4^+$ Count (cells/µl)	$CD8^+$ Count (cells/µl)
		**Donor A**		
A1	1992	900	ND[a]	1000
A3	1997	3000	900	1100
A4	1998	3000	1000	1100
A5	1998	5000	1000	1000
A6	2000	7000	1100	1400
		**Recipient B**		
B2	1992	ND	ND	ND
B3	1992	ND	200	650
B5	1998	500	275	375
B6	1999	300	330	375
		**Recipient C**		
C1	1992	ND	400	780
C2	1993	ND	190	700
C3	1994	ND	50	800
C4	1995	ND	30	525
C5	1996	80,000	20	250
C6	1996	150,000	10	300
C7	1997	115,000	10	200
C8	1997	35,000	10	450
C9	1998	42,000	10	320
C10	1998	50,000	10	275
C11	2000	80,000	10	250
C12	2000	175,000	10	410

[a] ND: no data available.
